# Poly[[μ-1,3-bis­(pyridin-3-yl)urea]bis­(μ_4_-glutarato)dicopper(II)]

**DOI:** 10.1107/S2414314623008337

**Published:** 2023-09-26

**Authors:** Frederick C. Ezenyilimba, Robert L. LaDuca

**Affiliations:** a E-35 Holmes Hall, Michigan State University, Lyman Briggs College, 919 E. Shaw Lane, East Lansing, MI 48825, USA; University of Kentucky, USA

**Keywords:** crystal structure, copper, coordination polymer, tri-periodic

## Abstract

The title compound, [Cu_2_(C_5_H_6_O_4_)_2_(C_11_H_10_N_4_O)]_*n*_, contains square-pyramidally coordinated Cu^II^ ions linked by *anti-gauche* conformation glutarate (glu) ligands into [Cu_2_(glu)_2_]_*n*_ di-periodic coordination polymer layers with embedded [Cu_2_(OCO)_4_] paddlewheel clusters. In turn, the layer motifs are connected by 1,3-di(pyridin-3-yl)urea (3-dpu) linkers to form a [Cu_2_(glu)_2_(3-dpu)]_*n*_ tri-periodic coordination polymer network. Treating the [Cu_2_(OCO)_4_] clusters as 6-connected nodes reveals an underlying 4^12^6^3^ **pcu** topology according to *TOPOSPRO* software [Blatov *et al.* (2014 ▸). *Cryst. Growth Des.* **14**, 3576–3586].

## Abstract 2

A divalent copper tri-periodic coordination polymer with no co-crystallized species and underlying **pcu** topology, {[Cu_2_(glu)_2_(3-dpu)]_*n*_, was prepared and structurally characterized by single-crystal X-ray diffraction.

## Graphical abstract

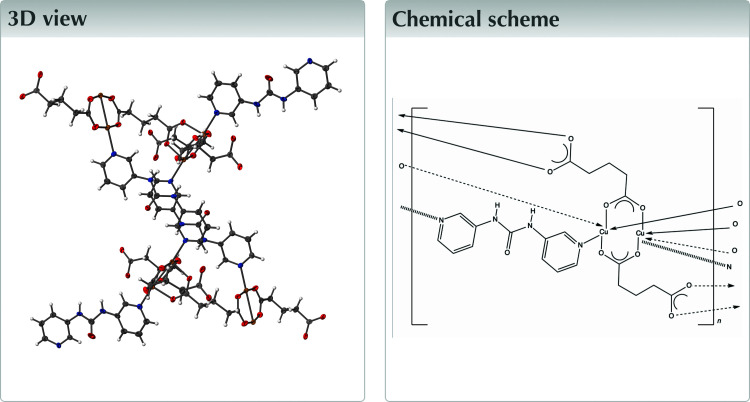

## Structure description

The conformationally flexible glutarate (glu) ligand has been used in our group previously to generate divalent copper coordination polymers, whose resulting topologies depend greatly on the nature of a dipyridyl-type co-ligand (Martin *et al.*, 2008 ▸). Use of 1,4-bis­(pyridin-4-ylmeth­yl)piperazine (4-bpmp) generated the tri-periodic coordination polymer {[Cu_2_(glu)_2_(4-bpmp)]·4H_2_O}_*n*_, which adopted a rare self-penetrated 4^4^6^10^8 **mab** topology. Using the isomeric *N*-(pyridin-3-yl)nicotinamide (3-pna) and *N*-(pyridin-4-yl)nicotinamide (4-pna) ligands afforded the non-inter­penetrated (4,4) grid di-periodic coordination polymer {[Cu(glu)(3-pna)(H_2_O)]·H_2_O}_*n*_ and the twofold inter­penetrated (6,3) grid di-periodic coordination polymer {[Cu(glu)(4-pna)(H_2_O)]·H_2_O}_*n*_, respectively (Uebler *et al.*, 2013 ▸). The title compound was prepared during an effort to prepare divalent copper coordination polymers containing both glu and 1,3-di(pyridin-3-yl)urea (3-dpu) ligands.

**Figure 1:**
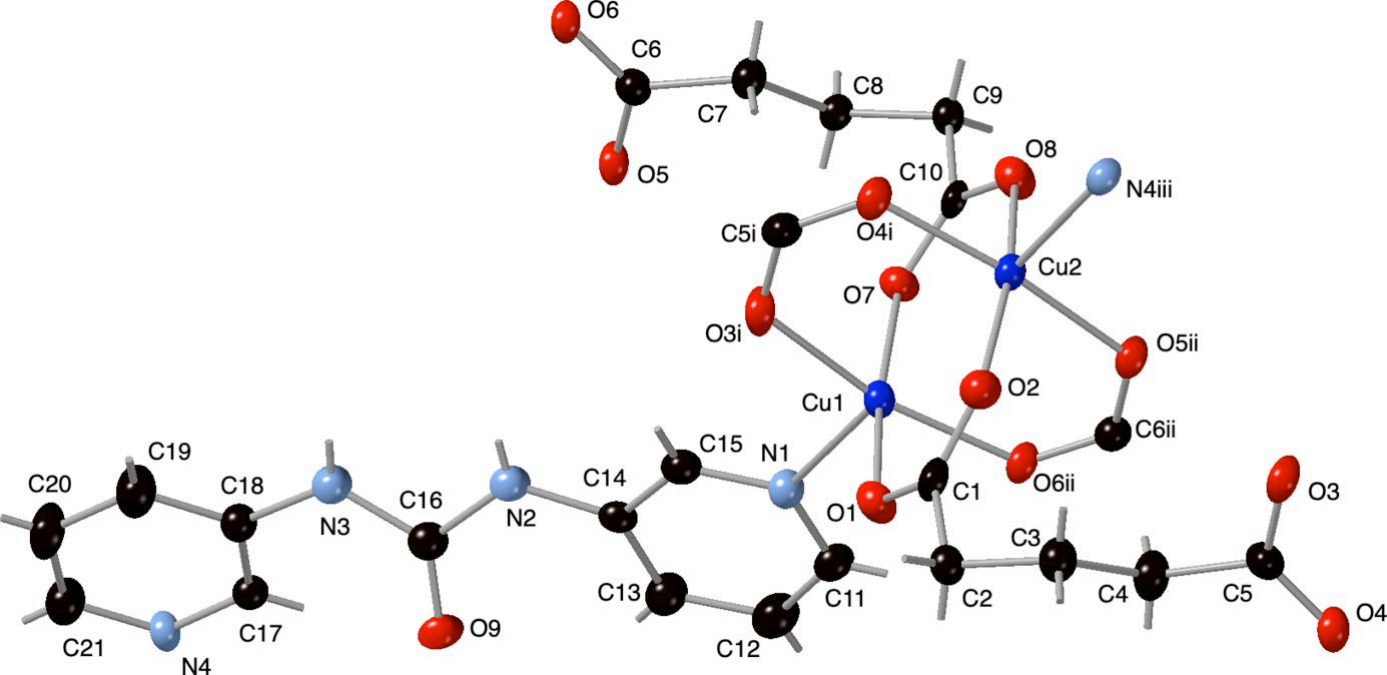
Copper coordination environments in the title compound with glu and 3-dpu ligands. Displacement ellipsoids are drawn at the 50% probability level. Color code: Cu, dark blue; O, red; N, light blue; C, black. H-atom positions are shown as gray sticks. Symmetry codes are as listed in Table 1 ▸.

The asymmetric unit of the title compound contains two divalent Cu atoms, two fully deprotonated glu ligands, and a 3-dpu ligand. The Cu^II^ atoms are both coordinated in an {O_4_N} square-pyramidal fashion (Fig. 1 ▸, Table 1 ▸) with a pyridyl N atom from a 3-dpu ligand in its Jahn–Teller-elongated axial positions. The basal planes of the coordination polyhedra around Cu^II^ are taken up by four O atoms belonging to different glu ligands. The bridging termini of the glu ligands form [Cu_2_(OCO)_4_] paddlewheel clusters with a Cu—Cu distance of 2.6512 (7) Å (Fig. 1 ▸). The crystallographically distinct glu ligands both adopt *anti-gauche* conformations [torsion angles = 59.9 (5) and 174.3 (3)°; 62.8 (4) and 171.9 (3)°.

**Figure 2:**
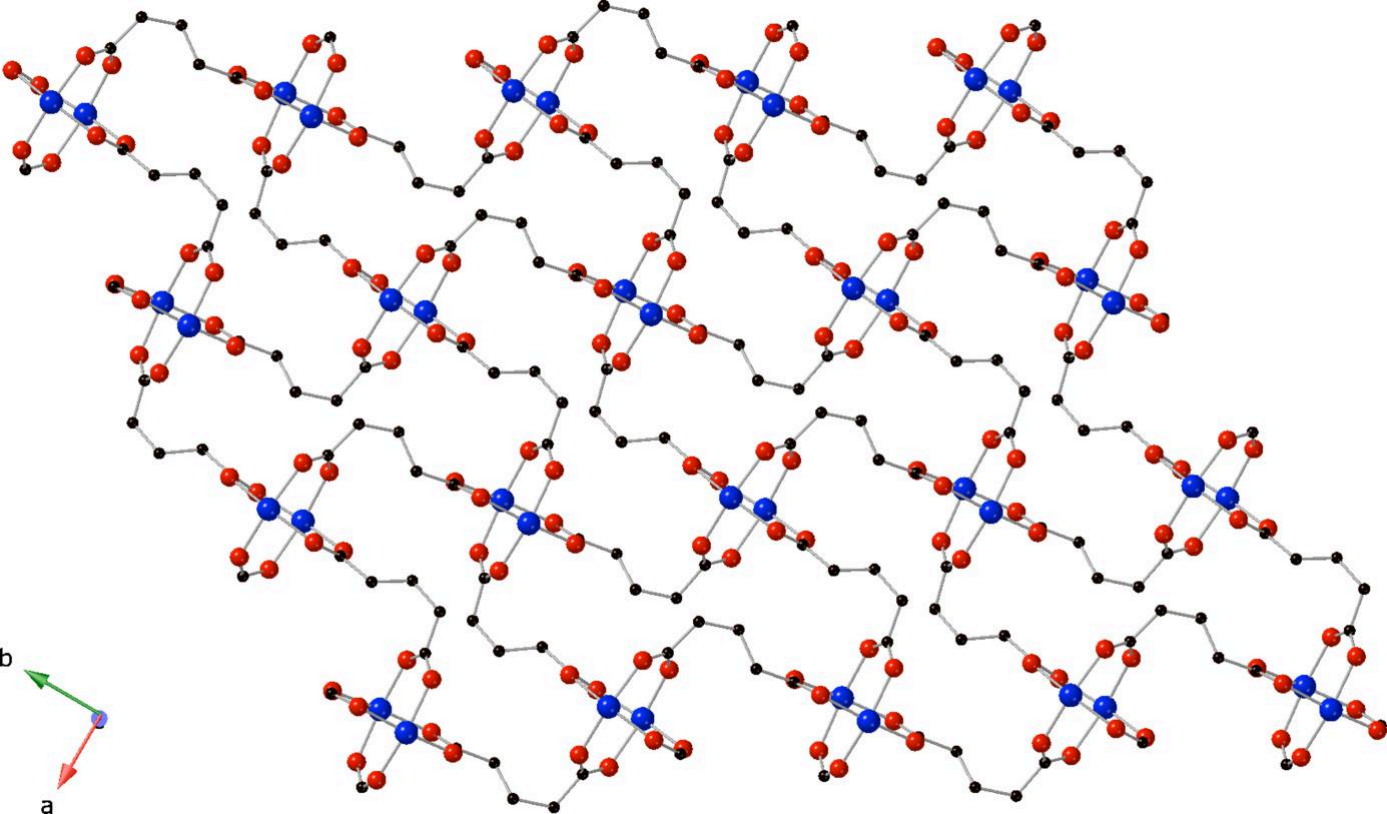
[Cu_2_(glu)_2_]_*n*_ layered motif in the title compound viewed in projection down the *a*-axis, featuring [Cu_2_(OCO)_4_] paddlewheel clusters.

**Figure 3:**
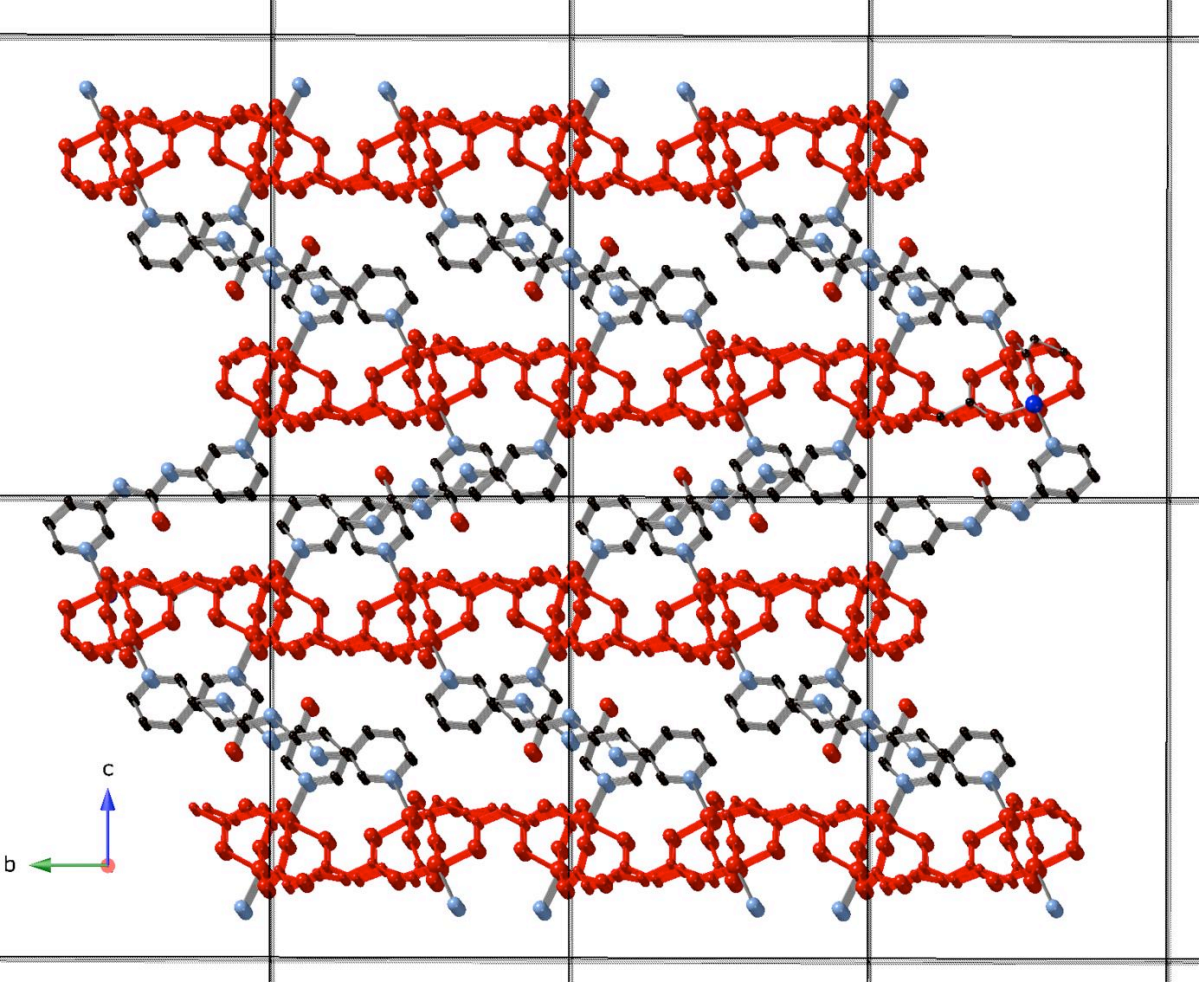
[Cu_2_(glu)_2_(3-dpu)]_*n*_ tri-periodic coordination polymer network in the title compound with unit cell outlines shown. [Cu_2_(glu)_2_]_*n*_ layered motifs are drawn in red.

**Figure 4:**
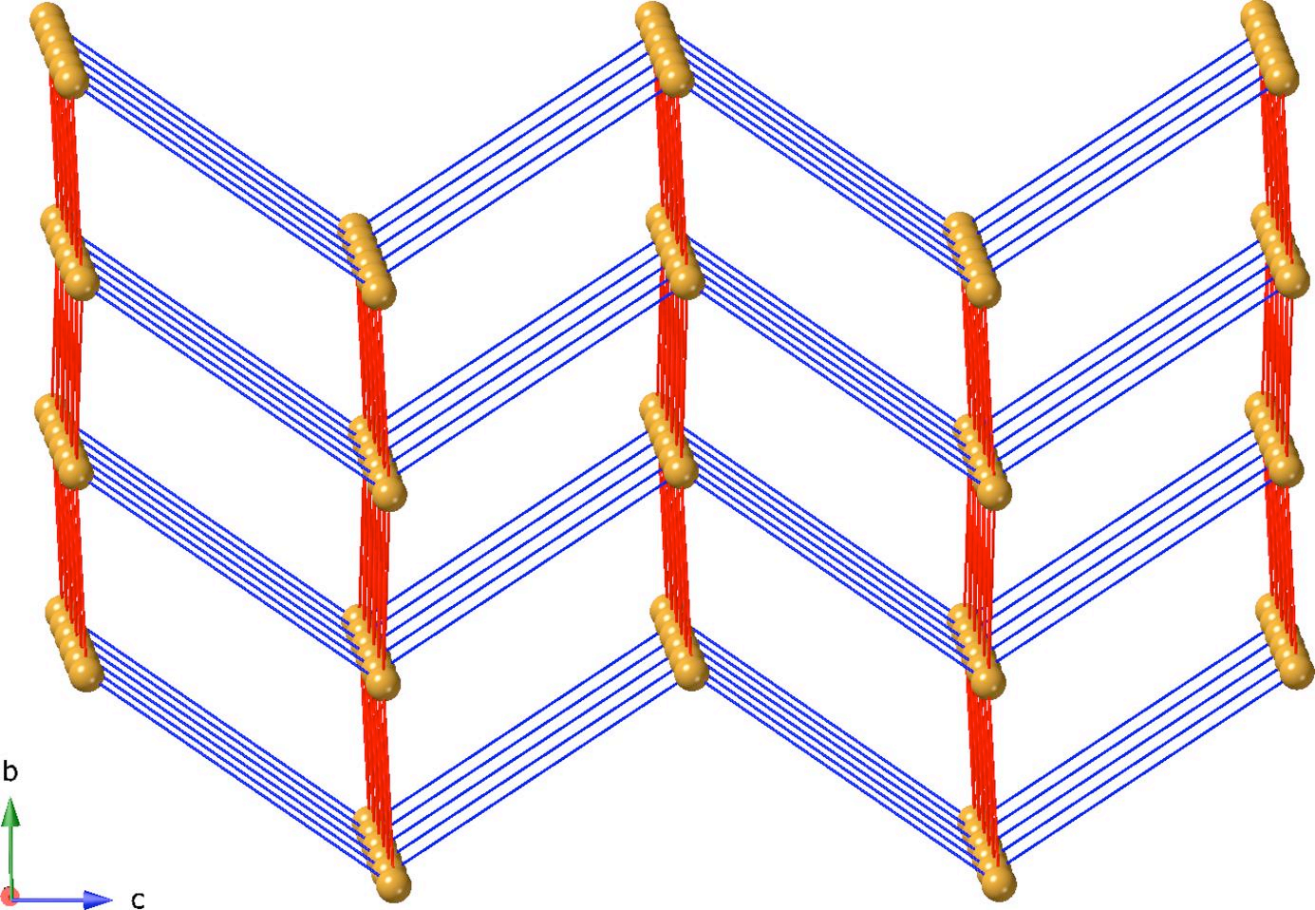
Schematic representation of the **pcu** network topology in the title compound. The centroids of the [Cu_2_(OCO)_4_] paddlewheel clusters are shown as gold spheres. The glu and 3-dpu ligand connections are shown as red rods and blue rods, respectively.

The full span of the glu ligands connects the [Cu_2_(OCO)_4_] paddlewheel clusters into di-periodic [Cu_2_(glu)_2_]_*n*_ coordination polymer layers that are oriented parallel to the *ab* crystallographic plane (Fig. 2 ▸). These layer motifs are pillared into a tri-periodic non-inter­penetrated [Cu_2_(glu)_2_(3-dpu)]_*n*_ coordination polymer network by 3-dpu ligands that span a Cu⋯Cu distance of 11.970 (1) Å (Fig. 3 ▸). Hydrogen-bonding inter­actions between the N—H moieties of the 3-dpu ligand and ligated carboxyl­ate O atoms (O8) of the glu ligands stabilize the tri-periodic network (Table 2 ▸). Treating the [Cu_2_(OCO)_4_] paddlewheel clusters as 6-connected nodes reveals an underlying 4^12^6^3^ **pcu** topology (Fig. 4 ▸).

## Synthesis and crystallization

Cu(NO_3_)_2_·2.5H_2_O (86 mg, 0.37 mmol), glutaric acid (gluH_2_) (50 mg, 0.37 mmol), 1,3-di(pyridin-3-yl)urea (3-dpu) (79 mg, 0.37 mmol), and 0.75 ml of a 1.0 *M* NaOH solution were placed into 10 ml distilled H_2_O in a Teflon-lined acid digestion bomb. The bomb was sealed and heated in an oven at 373 K for 24 h, and then cooled slowly to 273 K. Green crystals of the title complex were obtained in 58% yield.

## Refinement

Crystal data, data collection and structure refinement details are summarized in Table 3 ▸.

## Supplementary Material

Crystal structure: contains datablock(s) I, 1R. DOI: 10.1107/S2414314623008337/pk4043sup1.cif

Structure factors: contains datablock(s) I. DOI: 10.1107/S2414314623008337/pk4043Isup2.hkl

CCDC reference: 2296561

Additional supporting information:  crystallographic information; 3D view; checkCIF report

## Figures and Tables

**Table 1:** Selected geometric parameters (Å, °)

Cu1—O1	1.967 (3)	Cu2—O2	1.981 (3)
Cu1—O3^i^	2.000 (3)	Cu2—O4^i^	1.954 (3)
Cu1—O6^ii^	1.992 (3)	Cu2—O5^ii^	1.950 (3)
Cu1—O7	1.973 (2)	Cu2—O8	2.001 (3)
Cu1—N1	2.167 (3)	Cu2—N4^iii^	2.194 (3)
			
O1—Cu1—O3^i^	91.61 (12)	O2—Cu2—O8	164.87 (11)
O1—Cu1—O6^ii^	87.72 (11)	O2—Cu2—N4^iii^	93.03 (11)
O1—Cu1—O7	171.10 (11)	O4^i^—Cu2—O2	92.11 (11)
O1—Cu1—N1	100.51 (11)	O4^i^—Cu2—O8	86.16 (11)
O3^i^—Cu1—N1	99.46 (12)	O4^i^—Cu2—N4^iii^	93.08 (11)
O6^ii^—Cu1—O3^i^	165.11 (11)	O5^ii^—Cu2—O2	89.12 (11)
O6^ii^—Cu1—N1	95.28 (12)	O5^ii^—Cu2—O4^i^	169.86 (11)
O7—Cu1—O3^i^	88.61 (11)	O5^ii^—Cu2—O8	90.03 (11)
O7—Cu1—O6^ii^	89.78 (11)	O5^ii^—Cu2—N4^iii^	96.90 (11)
O7—Cu1—N1	88.22 (11)	O8—Cu2—N4^iii^	102.07 (11)

Symmetry codes: (i) 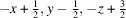 ; (ii) 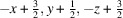 ; (iii) 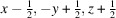 .

**Table 2:** Hydrogen-bond geometry (Å, °)

*D*—H⋯*A*	*D*—H	H⋯*A*	*D*⋯*A*	*D*—H⋯*A*
N2—H2⋯O8^iv^	0.88	1.93	2.767 (4)	157
N3—H3⋯O8^iv^	0.88	2.35	3.087 (4)	142

Symmetry code: (iv) 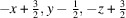 .

**Table 3:** Experimental details

Crystal data	
Chemical formula	[Cu_2_(C_5_H_6_O_4_)_2_(C_11_H_10_N_4_O)]
*M*_r_	601.50
Crystal system, space group	Monoclinic, *P*2_1_/*n*
Temperature (K)	173
*a*, *b*, *c* (Å)	8.5042 (11), 13.3095 (17), 20.921 (3)
β (°)	101.348 (1)
*V* (Å^3^)	2321.7 (5)
*Z*	4
Radiation type	Mo *K*α
μ (mm^−1^)	1.89
Crystal size (mm)	0.22 × 0.13 × 0.10

Data collection	
Diffractometer	Bruker APEXII CCD
Absorption correction	Multi-scan (*SADABS*; Krause *et al.*, 2015 ▸)
*T*_min_, *T*_max_	0.669, 0.745
No. of measured, independent and observed [*I* > 2σ(*I*)] reflections	18339, 4229, 3163
*R*_int_	0.060
(sin θ/λ)_max_ (Å^−1^)	0.602

Refinement	
*R*[*F* ^2^ > 2σ(*F* ^2^)], *wR*(*F* ^2^), *S*	0.042, 0.109, 1.03
No. of reflections	4229
No. of parameters	325
H-atom treatment	H-atom parameters constrained
Δρ_max_, Δρ_min_ (e Å^−3^)	0.72, −0.60

Computer programs: *COSMO* (Bruker, 2009 ▸), *SAINT* (Bruker, 2014 ▸), *SHELXT* (Sheldrick, 2015*a* ▸), *SHELXL* (Sheldrick, 2015*b* ▸), *CrystalMaker* (Palmer, 2020 ▸), and *OLEX2* (Dolomanov *et al.*, 2009 ▸).

## full crystallographic data

### Crystal data

**Table:**

[Cu_2_(C_5_H_6_O_4_)_2_(C_11_H_10_N_4_O)]	*F*(000) = 1224
*M**_r_* = 601.50	*D*_x_ = 1.721 Mg m^−^^3^
Monoclinic, *P*2_1_/*n*	Mo *K*α radiation, λ = 0.71073 Å
*a* = 8.5042 (11) Å	Cell parameters from 5350 reflections
*b* = 13.3095 (17) Å	θ = 2.5–25.3°
*c* = 20.921 (3) Å	µ = 1.89 mm^−^^1^
β = 101.348 (1)°	*T* = 173 K
*V* = 2321.7 (5) Å^3^	Block, green
*Z* = 4	0.22 × 0.13 × 0.10 mm

### Data collection

**Table:**

Bruker APEXII CCD diffractometer	4229 independent reflections
Radiation source: sealed tube	3163 reflections with *I* > 2σ(*I*)
Graphite monochromator	*R*_int_ = 0.060
Detector resolution: 8.36 pixels mm^-1^	θ_max_ = 25.3°, θ_min_ = 1.8°
ω scans	*h* = −10→10
Absorption correction: multi-scan (SADABS; Krause *et al.*, 2015)	*k* = −15→16
*T*_min_ = 0.669, *T*_max_ = 0.745	*l* = −24→25
18339 measured reflections	

### Refinement

**Table:**

Refinement on *F*^2^	Primary atom site location: dual
Least-squares matrix: full	Hydrogen site location: inferred from neighbouring sites
*R*[*F*^2^ > 2σ(*F*^2^)] = 0.042	H-atom parameters constrained
*wR*(*F*^2^) = 0.109	*w* = 1/[σ^2^(*F*_o_^2^) + (0.0481*P*)^2^ + 1.6221*P*] where *P* = (*F*_o_^2^ + 2*F*_c_^2^)/3
*S* = 1.03	(Δ/σ)_max_ = 0.001
4229 reflections	Δρ_max_ = 0.72 e Å^−^^3^
325 parameters	Δρ_min_ = −0.60 e Å^−^^3^
0 restraints	

### Special details

**Table:**

Experimental. Data was collected using a BRUKER CCD (charge-coupled device) based diffractometer equipped with an Oxford low-temperature apparatus operating at 173 K. A suitable crystal was chosen and mounted on a nylon loop using Paratone oil. Data were measured using ω scans of 0.5° per frame for 30 s. The total number of images were based on results from the program COSMO where redundancy was expected to be 4 and completeness to 0.83Å to 100%. Cell parameters were retrieved using *APEX II* software and refined using *SAINT* on all observed reflections. Data reduction was performed using the *SAINT* software, which corrects for Lp. Scaling and absorption corrections were applied using *SADABS* multi-scan technique, supplied by George Sheldrick. The structure was solved by dual-space methods using the *SHELXT* program and refined by the least squares method on F^2^, *SHELXL*, incorporated in *Olex2*.
Geometry. All esds (except the esd in the dihedral angle between two l.s. planes) are estimated using the full covariance matrix. The cell esds are taken into account individually in the estimation of esds in distances, angles and torsion angles; correlations between esds in cell parameters are only used when they are defined by crystal symmetry. An approximate (isotropic) treatment of cell esds is used for estimating esds involving l.s. planes.
Refinement. The structure was refined by least-squares using version 2018/3 of *SHELXL* (Sheldrick, 2015b) incorporated in *Olex2* (Dolomanov *et al.*, 2009). All non-hydrogen atoms were refined anisotropically. Hydrogen atom positions were calculated geometrically and refined using the riding model, except for the hydrogen atom on the nitrogen atom which was found by difference-Fourier methods and refined isotropically.

### Fractional atomic coordinates and isotropic or equivalent isotropic displacement parameters (Å^2^)

**Table:**

	*x*	*y*	*z*	*U*_iso_*/*U*_eq_	
Cu1	0.51833 (5)	0.46750 (3)	0.69093 (2)	0.01968 (15)	
Cu2	0.47185 (5)	0.55687 (3)	0.79894 (2)	0.01879 (15)	
O1	0.2886 (3)	0.4836 (2)	0.65382 (13)	0.0266 (6)	
O2	0.2499 (3)	0.55198 (19)	0.74755 (13)	0.0248 (6)	
O3	0.0222 (3)	0.84023 (19)	0.76407 (14)	0.0290 (7)	
O4	0.0402 (3)	0.9183 (2)	0.67157 (13)	0.0282 (7)	
O5	0.9976 (3)	0.18497 (18)	0.74221 (13)	0.0266 (6)	
O6	0.9526 (3)	0.11028 (19)	0.83279 (13)	0.0264 (6)	
O7	0.7420 (3)	0.46407 (19)	0.73954 (12)	0.0223 (6)	
O8	0.7091 (3)	0.5446 (2)	0.82984 (13)	0.0266 (7)	
O9	0.7057 (4)	0.1263 (2)	0.45026 (13)	0.0437 (9)	
N1	0.6018 (4)	0.4047 (2)	0.60804 (15)	0.0235 (7)	
N2	0.7042 (4)	0.1563 (3)	0.55713 (15)	0.0290 (8)	
H2	0.707005	0.127120	0.595097	0.035*	
N3	0.7539 (4)	−0.0029 (2)	0.52353 (15)	0.0272 (8)	
H3	0.752940	−0.020425	0.564012	0.033*	
N4	0.8952 (4)	−0.1190 (2)	0.38504 (15)	0.0213 (7)	
C1	0.2035 (4)	0.5284 (3)	0.6882 (2)	0.0222 (9)	
C2	0.0334 (4)	0.5567 (3)	0.6570 (2)	0.0245 (9)	
H2A	−0.039149	0.501040	0.663455	0.029*	
H2B	0.026550	0.564775	0.609461	0.029*	
C3	−0.0253 (5)	0.6543 (3)	0.6844 (2)	0.0267 (9)	
H3A	−0.137370	0.667748	0.662406	0.032*	
H3B	−0.023492	0.645728	0.731569	0.032*	
C4	0.0800 (5)	0.7435 (3)	0.6744 (2)	0.0298 (10)	
H4A	0.193184	0.725572	0.692056	0.036*	
H4B	0.069537	0.754582	0.626932	0.036*	
C5	0.0429 (4)	0.8420 (3)	0.70553 (18)	0.0198 (8)	
C6	0.9644 (4)	0.1855 (3)	0.79894 (18)	0.0200 (8)	
C7	0.9325 (4)	0.2872 (3)	0.82592 (19)	0.0237 (9)	
H7A	0.958344	0.283487	0.874114	0.028*	
H7B	0.816469	0.301817	0.812897	0.028*	
C8	1.0251 (4)	0.3746 (3)	0.80450 (18)	0.0211 (8)	
H8A	1.011660	0.374029	0.756416	0.025*	
H8B	1.140653	0.366472	0.823249	0.025*	
C9	0.9671 (4)	0.4753 (3)	0.82633 (19)	0.0204 (8)	
H9A	0.979623	0.475401	0.874385	0.024*	
H9B	1.034981	0.529804	0.814258	0.024*	
C10	0.7939 (4)	0.4961 (3)	0.79597 (18)	0.0183 (8)	
C11	0.6422 (5)	0.4641 (3)	0.56210 (19)	0.0286 (10)	
H11	0.628070	0.534704	0.564579	0.034*	
C12	0.7044 (5)	0.4241 (3)	0.5107 (2)	0.0368 (11)	
H12	0.732802	0.467621	0.478814	0.044*	
C13	0.7252 (5)	0.3215 (3)	0.5058 (2)	0.0328 (10)	
H13	0.766286	0.293544	0.470630	0.039*	
C14	0.6838 (5)	0.2605 (3)	0.55413 (18)	0.0240 (9)	
C15	0.6222 (4)	0.3057 (3)	0.60408 (18)	0.0235 (9)	
H15	0.593448	0.264071	0.636822	0.028*	
C16	0.7203 (5)	0.0954 (3)	0.50550 (19)	0.0274 (9)	
C17	0.8596 (4)	−0.0510 (3)	0.42751 (18)	0.0224 (9)	
H17	0.882606	0.017838	0.421486	0.027*	
C18	0.7902 (5)	−0.0778 (3)	0.48003 (19)	0.0239 (9)	
C19	0.7588 (5)	−0.1777 (3)	0.4894 (2)	0.0353 (11)	
H19	0.712509	−0.198164	0.525082	0.042*	
C20	0.7964 (6)	−0.2481 (3)	0.4455 (2)	0.0405 (12)	
H20	0.776981	−0.317547	0.451056	0.049*	
C21	0.8624 (5)	−0.2156 (3)	0.3938 (2)	0.0323 (10)	
H21	0.885280	−0.263684	0.363304	0.039*	

### Atomic displacement parameters (Å^2^)

**Table:**

	*U*^11^	*U*^22^	*U*^33^	*U*^12^	*U*^13^	*U*^23^
Cu1	0.0195 (3)	0.0170 (3)	0.0230 (3)	0.00133 (19)	0.0054 (2)	−0.00298 (19)
Cu2	0.0198 (3)	0.0156 (3)	0.0222 (3)	0.00055 (19)	0.0071 (2)	0.00043 (19)
O1	0.0186 (14)	0.0299 (16)	0.0298 (16)	0.0036 (12)	0.0014 (12)	−0.0097 (13)
O2	0.0209 (14)	0.0278 (16)	0.0263 (16)	0.0039 (12)	0.0061 (12)	0.0004 (12)
O3	0.0356 (17)	0.0153 (15)	0.0383 (18)	0.0027 (12)	0.0126 (14)	0.0039 (12)
O4	0.0403 (17)	0.0177 (15)	0.0288 (16)	0.0006 (13)	0.0123 (13)	−0.0039 (13)
O5	0.0406 (17)	0.0150 (14)	0.0268 (16)	0.0012 (12)	0.0126 (13)	−0.0037 (12)
O6	0.0357 (16)	0.0146 (15)	0.0289 (16)	−0.0016 (12)	0.0068 (13)	−0.0029 (12)
O7	0.0187 (14)	0.0276 (16)	0.0199 (14)	0.0035 (11)	0.0023 (11)	−0.0037 (12)
O8	0.0194 (14)	0.0308 (17)	0.0295 (16)	0.0049 (12)	0.0042 (12)	−0.0090 (12)
O9	0.080 (2)	0.0320 (18)	0.0171 (16)	0.0251 (17)	0.0046 (16)	0.0016 (13)
N1	0.0268 (18)	0.0211 (18)	0.0239 (18)	0.0048 (14)	0.0079 (15)	−0.0017 (14)
N2	0.044 (2)	0.027 (2)	0.0167 (17)	0.0117 (16)	0.0086 (16)	−0.0003 (14)
N3	0.042 (2)	0.0256 (19)	0.0178 (17)	0.0054 (16)	0.0148 (15)	0.0005 (15)
N4	0.0242 (17)	0.0174 (17)	0.0237 (17)	0.0003 (13)	0.0078 (14)	−0.0056 (14)
C1	0.022 (2)	0.013 (2)	0.033 (2)	−0.0033 (16)	0.0097 (18)	0.0042 (17)
C2	0.022 (2)	0.021 (2)	0.029 (2)	0.0002 (16)	0.0010 (17)	−0.0038 (17)
C3	0.022 (2)	0.024 (2)	0.034 (2)	0.0012 (17)	0.0072 (18)	−0.0019 (18)
C4	0.031 (2)	0.021 (2)	0.040 (3)	0.0023 (18)	0.014 (2)	−0.0032 (19)
C5	0.0117 (18)	0.026 (2)	0.022 (2)	−0.0026 (16)	0.0054 (16)	−0.0031 (17)
C6	0.0151 (19)	0.022 (2)	0.023 (2)	−0.0032 (16)	0.0029 (16)	−0.0012 (17)
C7	0.026 (2)	0.019 (2)	0.027 (2)	0.0011 (17)	0.0095 (18)	−0.0001 (17)
C8	0.022 (2)	0.018 (2)	0.023 (2)	0.0011 (16)	0.0047 (16)	0.0013 (16)
C9	0.019 (2)	0.017 (2)	0.025 (2)	−0.0013 (15)	0.0029 (16)	−0.0017 (16)
C10	0.023 (2)	0.0101 (18)	0.024 (2)	−0.0015 (15)	0.0094 (17)	0.0038 (16)
C11	0.035 (2)	0.024 (2)	0.026 (2)	0.0072 (18)	0.0043 (19)	0.0038 (18)
C12	0.052 (3)	0.034 (3)	0.029 (2)	0.006 (2)	0.018 (2)	0.006 (2)
C13	0.047 (3)	0.028 (2)	0.026 (2)	0.006 (2)	0.014 (2)	0.0016 (19)
C14	0.029 (2)	0.026 (2)	0.016 (2)	0.0046 (18)	0.0012 (17)	−0.0011 (17)
C15	0.026 (2)	0.027 (2)	0.019 (2)	0.0012 (17)	0.0062 (17)	0.0015 (17)
C16	0.029 (2)	0.027 (2)	0.025 (2)	0.0079 (18)	0.0035 (18)	−0.0015 (19)
C17	0.026 (2)	0.021 (2)	0.020 (2)	0.0034 (16)	0.0038 (17)	0.0002 (16)
C18	0.026 (2)	0.022 (2)	0.023 (2)	0.0048 (17)	0.0069 (17)	−0.0015 (17)
C19	0.053 (3)	0.026 (2)	0.035 (3)	−0.002 (2)	0.027 (2)	−0.0012 (19)
C20	0.068 (3)	0.019 (2)	0.042 (3)	−0.002 (2)	0.029 (3)	0.000 (2)
C21	0.047 (3)	0.025 (2)	0.030 (2)	0.004 (2)	0.019 (2)	−0.0010 (19)

### Geometric parameters (Å, º)

**Table:**

Cu1—Cu2	2.6512 (7)	C2—C3	1.542 (5)
Cu1—O1	1.967 (3)	C3—H3A	0.9900
Cu1—O3^i^	2.000 (3)	C3—H3B	0.9900
Cu1—O6^ii^	1.992 (3)	C3—C4	1.527 (5)
Cu1—O7	1.973 (2)	C4—H4A	0.9900
Cu1—N1	2.167 (3)	C4—H4B	0.9900
Cu2—O2	1.981 (3)	C4—C5	1.525 (5)
Cu2—O4^i^	1.954 (3)	C6—C7	1.512 (5)
Cu2—O5^ii^	1.950 (3)	C7—H7A	0.9900
Cu2—O8	2.001 (3)	C7—H7B	0.9900
Cu2—N4^iii^	2.194 (3)	C7—C8	1.520 (5)
O1—C1	1.266 (4)	C8—H8A	0.9900
O2—C1	1.266 (5)	C8—H8B	0.9900
O3—C5	1.271 (4)	C8—C9	1.529 (5)
O4—C5	1.236 (5)	C9—H9A	0.9900
O5—C6	1.273 (4)	C9—H9B	0.9900
O6—C6	1.242 (4)	C9—C10	1.511 (5)
O7—C10	1.251 (4)	C11—H11	0.9500
O8—C10	1.280 (4)	C11—C12	1.394 (6)
O9—C16	1.210 (5)	C12—H12	0.9500
N1—C11	1.340 (5)	C12—C13	1.383 (6)
N1—C15	1.334 (5)	C13—H13	0.9500
N2—H2	0.8800	C13—C14	1.396 (5)
N2—C14	1.398 (5)	C14—C15	1.394 (5)
N2—C16	1.378 (5)	C15—H15	0.9500
N3—H3	0.8800	C17—H17	0.9500
N3—C16	1.376 (5)	C17—C18	1.392 (5)
N3—C18	1.424 (5)	C18—C19	1.378 (6)
N4—C17	1.344 (5)	C19—H19	0.9500
N4—C21	1.336 (5)	C19—C20	1.392 (6)
C1—C2	1.513 (5)	C20—H20	0.9500
C2—H2A	0.9900	C20—C21	1.384 (6)
C2—H2B	0.9900	C21—H21	0.9500
			
O1—Cu1—Cu2	89.12 (8)	C3—C4—H4B	108.3
O1—Cu1—O3^i^	91.61 (12)	H4A—C4—H4B	107.4
O1—Cu1—O6^ii^	87.72 (11)	C5—C4—C3	115.7 (3)
O1—Cu1—O7	171.10 (11)	C5—C4—H4A	108.3
O1—Cu1—N1	100.51 (11)	C5—C4—H4B	108.3
O3^i^—Cu1—Cu2	84.86 (8)	O3—C5—C4	118.4 (3)
O3^i^—Cu1—N1	99.46 (12)	O4—C5—O3	125.3 (4)
O6^ii^—Cu1—Cu2	80.26 (8)	O4—C5—C4	116.3 (3)
O6^ii^—Cu1—O3^i^	165.11 (11)	O5—C6—C7	116.2 (3)
O6^ii^—Cu1—N1	95.28 (12)	O6—C6—O5	125.8 (4)
O7—Cu1—Cu2	82.03 (7)	O6—C6—C7	118.0 (3)
O7—Cu1—O3^i^	88.61 (11)	C6—C7—H7A	108.4
O7—Cu1—O6^ii^	89.78 (11)	C6—C7—H7B	108.4
O7—Cu1—N1	88.22 (11)	C6—C7—C8	115.7 (3)
N1—Cu1—Cu2	169.27 (9)	H7A—C7—H7B	107.4
O2—Cu2—Cu1	78.96 (8)	C8—C7—H7A	108.4
O2—Cu2—O8	164.87 (11)	C8—C7—H7B	108.4
O2—Cu2—N4^iii^	93.03 (11)	C7—C8—H8A	109.3
O4^i^—Cu2—Cu1	82.61 (8)	C7—C8—H8B	109.3
O4^i^—Cu2—O2	92.11 (11)	C7—C8—C9	111.5 (3)
O4^i^—Cu2—O8	86.16 (11)	H8A—C8—H8B	108.0
O4^i^—Cu2—N4^iii^	93.08 (11)	C9—C8—H8A	109.3
O5^ii^—Cu2—Cu1	87.76 (8)	C9—C8—H8B	109.3
O5^ii^—Cu2—O2	89.12 (11)	C8—C9—H9A	109.1
O5^ii^—Cu2—O4^i^	169.86 (11)	C8—C9—H9B	109.1
O5^ii^—Cu2—O8	90.03 (11)	H9A—C9—H9B	107.9
O5^ii^—Cu2—N4^iii^	96.90 (11)	C10—C9—C8	112.3 (3)
O8—Cu2—Cu1	85.91 (8)	C10—C9—H9A	109.1
O8—Cu2—N4^iii^	102.07 (11)	C10—C9—H9B	109.1
N4^iii^—Cu2—Cu1	170.71 (8)	O7—C10—O8	124.2 (3)
C1—O1—Cu1	117.5 (2)	O7—C10—C9	117.9 (3)
C1—O2—Cu2	128.3 (2)	O8—C10—C9	117.9 (3)
C5—O3—Cu1^iv^	120.4 (2)	N1—C11—H11	119.4
C5—O4—Cu2^iv^	126.1 (3)	N1—C11—C12	121.2 (4)
C6—O5—Cu2^v^	119.3 (2)	C12—C11—H11	119.4
C6—O6—Cu1^v^	126.8 (3)	C11—C12—H12	119.7
C10—O7—Cu1	127.1 (2)	C13—C12—C11	120.5 (4)
C10—O8—Cu2	120.3 (2)	C13—C12—H12	119.7
C11—N1—Cu1	121.2 (3)	C12—C13—H13	121.1
C15—N1—Cu1	119.8 (3)	C12—C13—C14	117.8 (4)
C15—N1—C11	118.9 (3)	C14—C13—H13	121.1
C14—N2—H2	117.3	C13—C14—N2	124.3 (4)
C16—N2—H2	117.3	C15—C14—N2	117.2 (3)
C16—N2—C14	125.3 (3)	C15—C14—C13	118.5 (4)
C16—N3—H3	118.3	N1—C15—C14	123.1 (4)
C16—N3—C18	123.4 (3)	N1—C15—H15	118.5
C18—N3—H3	118.3	C14—C15—H15	118.5
C17—N4—Cu2^vi^	115.5 (3)	O9—C16—N2	122.8 (4)
C21—N4—Cu2^vi^	125.3 (3)	O9—C16—N3	124.2 (4)
C21—N4—C17	118.5 (3)	N3—C16—N2	113.0 (3)
O1—C1—C2	118.5 (3)	N4—C17—H17	118.8
O2—C1—O1	124.9 (4)	N4—C17—C18	122.3 (4)
O2—C1—C2	116.7 (3)	C18—C17—H17	118.8
C1—C2—H2A	108.8	C17—C18—N3	120.3 (4)
C1—C2—H2B	108.8	C19—C18—N3	120.8 (4)
C1—C2—C3	113.6 (3)	C19—C18—C17	118.9 (4)
H2A—C2—H2B	107.7	C18—C19—H19	120.7
C3—C2—H2A	108.8	C18—C19—C20	118.7 (4)
C3—C2—H2B	108.8	C20—C19—H19	120.7
C2—C3—H3A	109.4	C19—C20—H20	120.4
C2—C3—H3B	109.4	C21—C20—C19	119.1 (4)
H3A—C3—H3B	108.0	C21—C20—H20	120.4
C4—C3—C2	111.2 (3)	N4—C21—C20	122.4 (4)
C4—C3—H3A	109.4	N4—C21—H21	118.8
C4—C3—H3B	109.4	C20—C21—H21	118.8
C3—C4—H4A	108.3		
			
Cu1—O1—C1—O2	−9.7 (5)	N4—C17—C18—C19	0.9 (6)
Cu1—O1—C1—C2	170.2 (2)	C1—C2—C3—C4	59.9 (5)
Cu1^iv^—O3—C5—O4	−0.2 (5)	C2—C3—C4—C5	−174.3 (3)
Cu1^iv^—O3—C5—C4	177.6 (2)	C3—C4—C5—O3	46.1 (5)
Cu1^v^—O6—C6—O5	0.0 (5)	C3—C4—C5—O4	−135.9 (4)
Cu1^v^—O6—C6—C7	178.2 (2)	C6—C7—C8—C9	171.9 (3)
Cu1—O7—C10—O8	−5.9 (5)	C7—C8—C9—C10	−62.8 (4)
Cu1—O7—C10—C9	173.4 (2)	C8—C9—C10—O7	−33.2 (5)
Cu1—N1—C11—C12	176.3 (3)	C8—C9—C10—O8	146.2 (3)
Cu1—N1—C15—C14	−176.4 (3)	C11—N1—C15—C14	0.0 (6)
Cu2—O2—C1—O1	15.3 (5)	C11—C12—C13—C14	−0.8 (7)
Cu2—O2—C1—C2	−164.6 (2)	C12—C13—C14—N2	−177.3 (4)
Cu2^iv^—O4—C5—O3	7.5 (5)	C12—C13—C14—C15	0.8 (6)
Cu2^iv^—O4—C5—C4	−170.3 (2)	C13—C14—C15—N1	−0.4 (6)
Cu2^v^—O5—C6—O6	1.9 (5)	C14—N2—C16—O9	−6.0 (7)
Cu2^v^—O5—C6—C7	−176.4 (2)	C14—N2—C16—N3	174.2 (3)
Cu2—O8—C10—O7	8.7 (5)	C15—N1—C11—C12	0.0 (6)
Cu2—O8—C10—C9	−170.6 (2)	C16—N2—C14—C13	−20.8 (6)
Cu2^vi^—N4—C17—C18	171.7 (3)	C16—N2—C14—C15	161.2 (4)
Cu2^vi^—N4—C21—C20	−172.1 (3)	C16—N3—C18—C17	27.5 (6)
O1—C1—C2—C3	−147.7 (3)	C16—N3—C18—C19	−153.1 (4)
O2—C1—C2—C3	32.2 (5)	C17—N4—C21—C20	−1.4 (6)
O5—C6—C7—C8	−32.1 (5)	C17—C18—C19—C20	−0.6 (6)
O6—C6—C7—C8	149.5 (3)	C18—N3—C16—O9	6.1 (6)
N1—C11—C12—C13	0.4 (7)	C18—N3—C16—N2	−174.1 (3)
N2—C14—C15—N1	177.8 (3)	C18—C19—C20—C21	−0.6 (7)
N3—C18—C19—C20	180.0 (4)	C19—C20—C21—N4	1.6 (7)
N4—C17—C18—N3	−179.7 (3)	C21—N4—C17—C18	0.1 (6)

Symmetry codes: (i) −*x*+1/2, *y*−1/2, −*z*+3/2; (ii) −*x*+3/2, *y*+1/2, −*z*+3/2; (iii) *x*−1/2, −*y*+1/2, *z*+1/2; (iv) −*x*+1/2, *y*+1/2, −*z*+3/2; (v) −*x*+3/2, *y*−1/2, −*z*+3/2; (vi) *x*+1/2, −*y*+1/2, *z*−1/2.

### Hydrogen-bond geometry (Å, º)

**Table:**

*D*—H···*A*	*D*—H	H···*A*	*D*···*A*	*D*—H···*A*
N2—H2···O8^v^	0.88	1.93	2.767 (4)	157
N3—H3···O8^v^	0.88	2.35	3.087 (4)	142
C13—H13···O9	0.95	2.31	2.837 (5)	115
C17—H17···O4^vii^	0.95	2.33	2.973 (5)	124
C17—H17···O9	0.95	2.25	2.784 (5)	115

Symmetry codes: (v) −*x*+3/2, *y*−1/2, −*z*+3/2; (vii) −*x*+1, −*y*+1, −*z*+1.
